# Homeostatic plasticity and synaptic scaling in the adult mouse auditory cortex

**DOI:** 10.1038/s41598-017-17711-5

**Published:** 2017-12-12

**Authors:** Manuel Teichert, Lutz Liebmann, Christian A. Hübner, Jürgen Bolz

**Affiliations:** 1 University of Jena, Institute of General Zoology and Animal Physiology, 07743 Jena, Germany; 2 University of Jena, University Hospital Jena, Institute of Human Genetics, 07743 Jena, Germany

## Abstract

It has been demonstrated that sensory deprivation results in homeostatic adjustments recovering neuronal activity of the deprived cortex. For example, deprived vision multiplicatively scales up mEPSC amplitudes in the primary visual cortex, commonly referred to as synaptic scaling. However, whether synaptic scaling also occurs in auditory cortex after auditory deprivation remains elusive. Using periodic intrinsic optical imaging in adult mice, we show that conductive hearing loss (CHL), initially led to a reduction of primary auditory cortex (A1) responsiveness to sounds. However, this was followed by a complete recovery of A1 activity evoked sounds above the threshold for bone conduction, 3 days after CHL. Over the same time course patch-clamp experiments in slices revealed that mEPSC amplitudes in A1 layers 2/3 pyramids scaled up multiplicatively in CHL mice. No recovery of sensory evoked A1 activation was evident in TNFα KO animals, which lack synaptic scaling. Additionally, we could show that the suppressive effect of sounds on visually evoked visual cortex activity completely recovered along with TNFα dependent A1 homeostasis in WT animals. This is the first demonstration of homeostatic multiplicative synaptic scaling in the adult A1. These findings suggest that mild hearing loss massively affects auditory processing in adult A1.

## Introduction

Homeostatic plasticity is essential in maintaining a stable firing rate in neurons to compensate for prolonged perturbations of neuronal activity^1,2^. For example, a prolonged reduction of neuronal activity of cultured neocortical neurons generated compensatory changes returning firing levels back to control values^1^. Such homeostatic regulations are typically accompanied by an additional insertion of 2-amino-3-(3-hydroxy-5-methyl-isoxasol-4-yl)propanoic acid receptors (AMPARs) into all synapses of a neuron, leading to multiplicatively scaled up mEPSC (miniature excitatory postsynaptic currents) amplitudes or, “synaptic scaling”^2,3^. Specifically, this mechanism is described to globally increase (or decrease) the strength of each synapse of a neuron by the same factor in a multiplicative manner which allows the conservation of information stored in the synaptic weights^4^. Previous studies have demonstrated that synaptic scaling also takes place *in vivo*, e.g. after reduction of sensory cortex activity due to sensory deprivation. For example, visual deprivation by intraocular TTX injections, dark exposure, binocular enucleation or retinal lesions scales up AMPAR mediated mEPSC amplitudes in layers 2/3 pyramids of the primary visual cortex (V1) in juvenile and adult mice^5–9^. Furthermore, it was shown that prolonged visual deprivation increases visually evoked V1 responses during the visual critical period^10^. However, evidence for homeostatic synaptic scaling in primary auditory cortex and its effects on cortical responsiveness following auditory deprivation is rare.

Previously, two models for induction of an auditory deprivation have been used, sensorineural hearing loss (SNHL)^11–13^ and conductive hearing loss (CHL)^14–17^. Whereas SNHL removes both sensory evoked activity and spontaneous activity arising from the organ of Corti^3^, CHL only reduces sound transmission to the inner ear without affecting the cochlea, reflecting a milder form of hearing loss^11^. CHL is of great clinical relevance, since it represents the second most common form of hearing loss^17,18^ and affects individuals across all ages^19,20^. Recent studies investigated the effects of CHL on early processing structures of the auditory pathway^16–18,21^ or on developing auditory cortex^11,15,22,23^. For example, CHL upregulated subunits of AMPARs in the cochlear nucleus^17,18^, suggesting a homeostatic compensatory mechanism to a reduced afferent input, but did not provoke synaptic scaling at this early stage of auditory pathway^16^. However, the effects of CHL on adult A1 are poorly understood.

Here, we investigated the effects of a prolonged bilateral CHL, induced by bilateral malleus removal, on stimulus evoked responsiveness and single cell properties in the matured A1. Using optical imaging of intrinsic signals, we could show that sound evoked A1 activity dramatically decreased after CHL. Notably, this reduction was followed by a complete recovery of A1 responsiveness, elicited by supra-threshold low frequency stimuli, back to control levels 3 d after CHL. By performing electrophysiological single cell recordings in brain slices obtained 3 d after CHL, we found amplitudes of spontaneous mEPSCs of A1 layers 2/3 neurons to be increased multiplicatively, strongly indicating homeostatic synaptic scaling. In agreement with this interpretation, sound evoked A1 responsiveness did not recover in TNFα KO animals, which have previously been shown to lack synaptic scaling *in vitro* and *in vivo* ^10,24^. Furthermore, using intrinsic imaging we could demonstrate that cross-modal effects of A1 on V1 disappeared directly after CHL, but completely recovered over the subsequent 3 d in WT animals. Such a recovery was, however, absent in TNFαKO mice. Thus, our data strongly suggest that CHL provokes homeostatic plasticity in form of synaptic scaling in A1, which profoundly contributes to the recovery of stimulus evoked A1 responsiveness and cross-modal A1-V1 interactions. Thus, the present study highlights the ability of adult A1 to undergo homeostatic changes in response to mild hearing loss.

## Results

### Fourier based periodic intrinsic imaging of the auditory cortex

**Figure 1:**
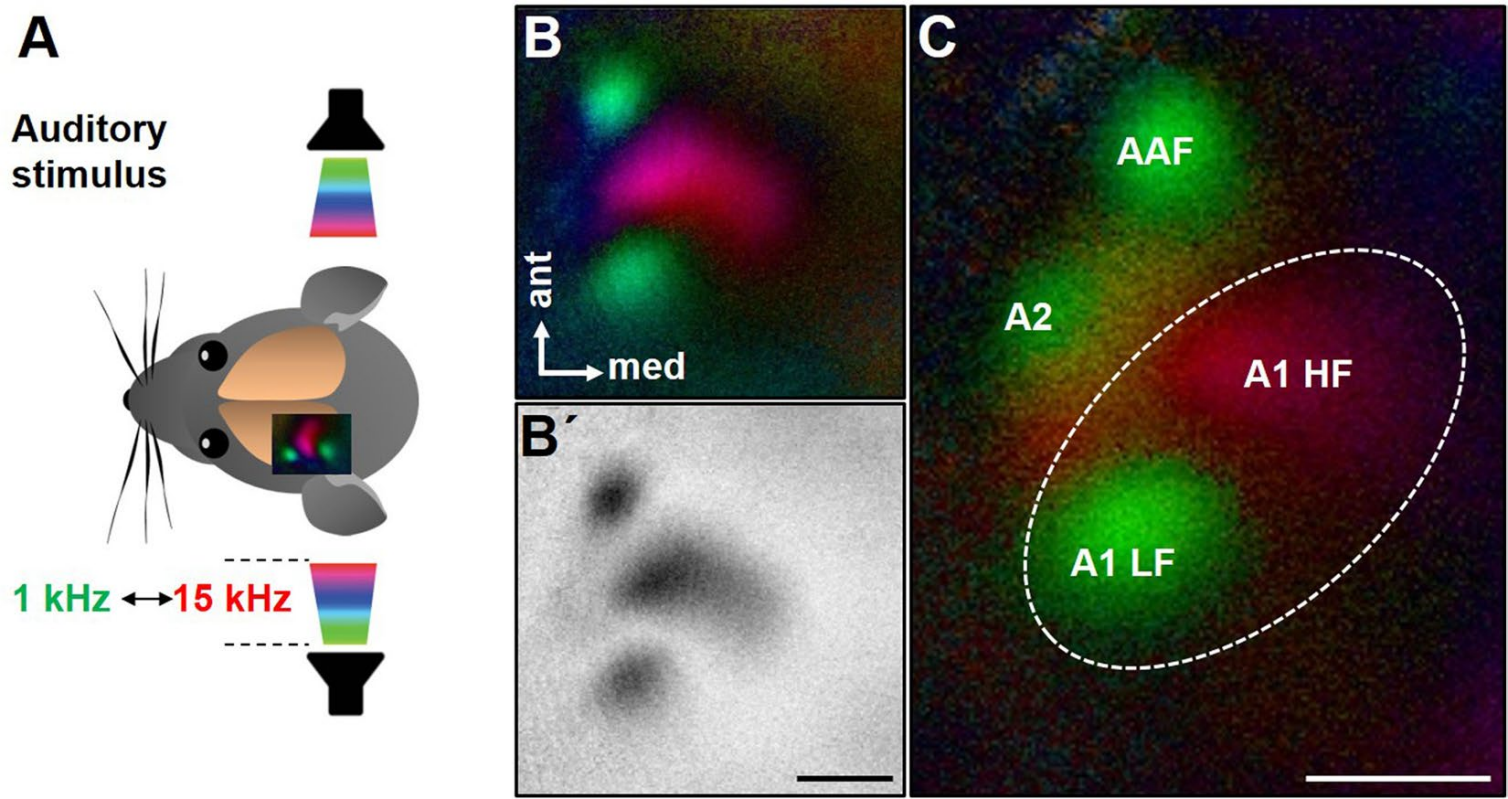
Intrinsic optical imaging of the auditory cortex. **(A)** Tone sweeps ranging from 1 kHz to 15 kHz at 70 dB SPL were delivered by free field speakers placed 15 cm next to both ears. **(B**, **B′**) Representative polar map and amplitude map mouse of the auditory cortex. **(C)** Organization of the auditory cortex: AAF: anterior auditory field, A2: secondary auditory cortex, A1LF: Low frequency region of primary auditory cortex, A1HF: High frequency region of primary auditory cortex. Scale bar: 500 µm.

As a first step we designed an auditory stimulus that enabled us to generate reliable tonotopic maps of the auditory cortex in mice using Fourier intrinsic optical imaging. This technique allows non-invasive measurements of stimulus evoked cortical responses in the visual^10,25–28^ and auditory cortex^29,30^ and its reliability has been validated by electrophysiological recordings^10,31^. Because intrinsic optical imaging is based on temporally periodic stimulus presentation^25^, we used tone sweeps linearly ascending or descending in frequency (1–15 kHz) with 70 dB sound pressure level (SPL), which were repeated with a period of 8 s for 5 min. As depicted in Fig. 1A, the auditory stimuli were delivered by free field speakers placed next to both ears. In order to eliminate the hemodynamic delay of intrinsic signals, we reversed both stimuli. The auditory evoked cortical responses were then recorded through the intact scull of the left hemisphere in adult mice (Fig. 1A).

Tonotopic maps of the mouse auditory cortex are shown in Fig. 1B and C and a representative magnitude map is illustrated in Fig. 1B′. It is clearly visible that the auditory cortex response appears in three to four spatially distinct areas. Specifically, the green loci of the polar maps (Fig. 1B,C) represent auditory fields responding to low frequency stimuli ranging from 1 kHz to about 5 kHz. In contrast, stimuli with higher frequencies evoked cortical responses in the red colored area. According to recent studies investigating the organization of mouse auditory cortex^30,32,33^, the two to three green areas represent the low frequency field of A1 (A1 LF), the secondary auditory cortex (A2), and the anterior auditory field (AAF). The red area represents the high frequency field of A1 (A1 HF). In all animals tested (n = 20) we always detected A1 LF, A1 HF and the AAF, whereas A2 was only detected in 7 animals.

The goal of the present study was to investigate the effects of CHL on auditory cortex activity in fully adult mice far beyond their sensory critical periods. Hence, we decided to use animals with an age of 90 to 150 days (3 to 5 month) for this study. It has been described that C57BL/6 J mice display the classic pattern of age-related hearing loss (AHL) at an age of 12 to 15 month^34,35^. However, there is also some evidence of slight hearing threshold shifts already at 4 month of age^36^.

To investigate whether sound evoked A1 activity levels might be influenced by an early onset AHL, we first measured A1 responsiveness to sound sweeps of 60 dB and 70 dB SPL in normal mice between 3 and 5 month of age using intrinsic imaging. Our quantification revealed that there was no correlation between the age of the animals and the sound evoked response amplitudes of A1 (A1 LF, 60 dB: R squared = 0.06, p = 0.68; A1 HF, 60 dB, R squared = 0.03, p = 0.79; A1 LF, 70 dB: R squared = 0.09, p = 0.57; A1 HF, 70 dB: R squared = 0.02, p = 0.79; Supplementary figure 1A–D). Hence, in the age range examined here, 90 to 150 days, A1 responsiveness to the delivered sound seeps is consistent, and thus, not affected by the age.

### A1 responsiveness recovers 3 d after auditory deprivation

**Figure 2:**
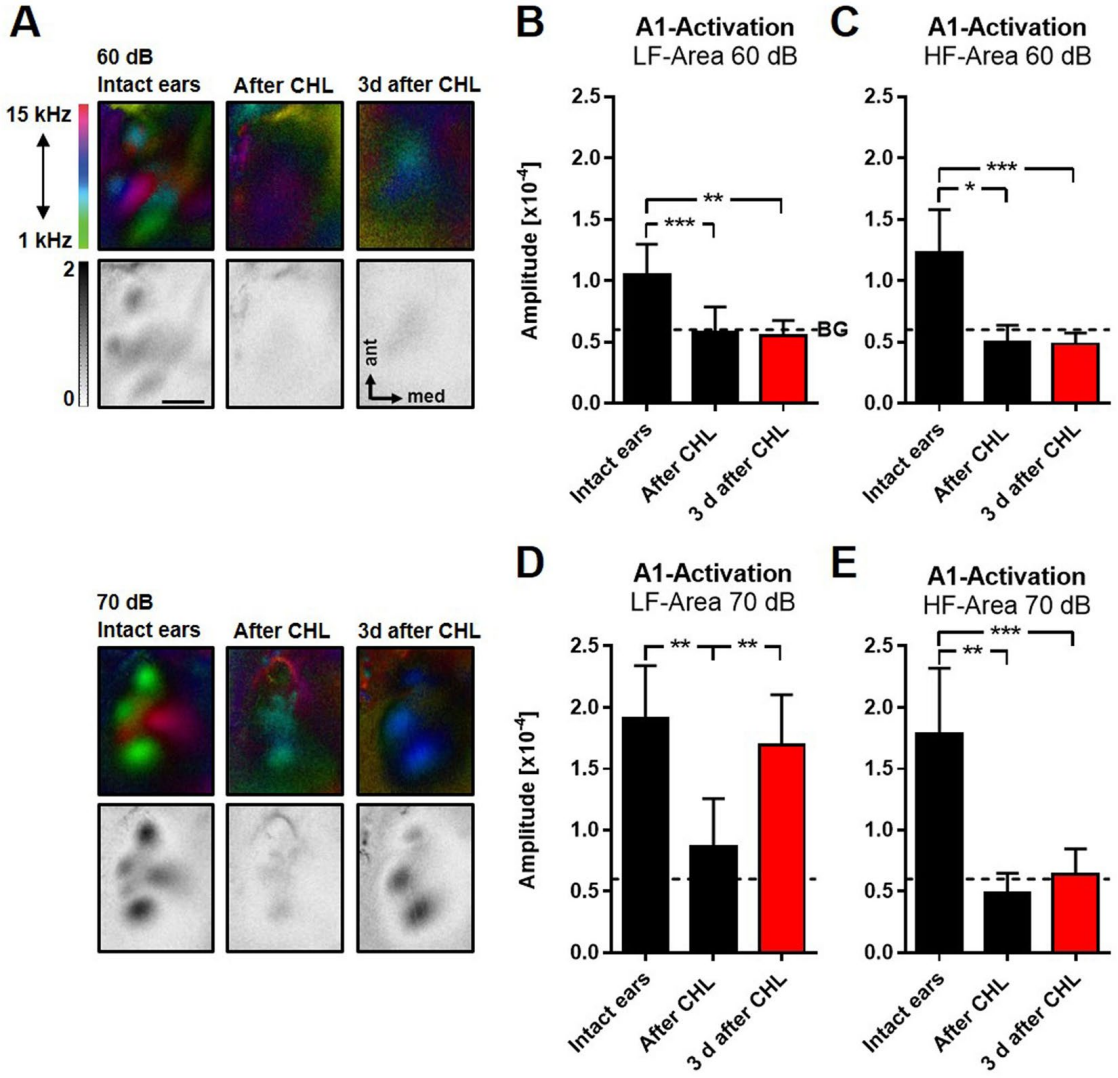
Intrinsic optical imaging of auditory cortex in mice with intact ears, immediately after and 3 d after CHL. **(A)** Representative color coded polar maps and corresponding grey scale coded amplitude maps of the auditory cortex evoked by auditory stimulation with 60 dB and 70 dB before and at different time points after CHL. (**B,C**) Responsiveness of the A1 LF and A1 HF region of A1 evoked by sound sweeps of 60 dB SPL decreased to background level directly after CHL and remained unchanged 3 d after CHL (intact ears and after CHL: n = 5; 3 d after CHL: n = 6). (**D,E**) The activation of LF and HF regions evoked by sounds of 70 dB also markedly decreased after CHL (n = 6). However, 3 d after CHL there was a recovery of A1 LF responsiveness, which was not detected in the A1 HF region (n = 6). Data are presented as means ± SD.; *p < 0.05, **p < 0.01, ***p < 0.001; BG: background activity level; Scale bar: 500 µm.

Next, we investigated whether activity levels in the adult auditory cortex adjust *in vivo* after CHL induced by bilateral malleus removal. We measured A1 responsiveness to sound sweeps of 60 dB and 70 dB SPL in the same animals with intact ears and immediately, i.e. 10 min to 20 min after CHL, and in an additional group of mice 3 d after CHL using intrinsic signal imaging. Figure 2A depicts representative tonotopic polar maps and the corresponding grey scaled amplitude maps of the auditory cortex evoked by sound sweeps of 60 dB and 70 dB SPL in animals with intact ears, directly after and 3 d after CHL. It is clearly visible that the activity patches evoked by sounds of all SPLs became less visible immediately after CHL, indicating a substantial reduction of A1 responsiveness to sounds. This also indicates that the method used for inducing a CHL was effective and reliable. However, while the patches evoked by 60 dB stimulation were completely abolished after CHL, there was always a weak residual activation of the auditory cortex to 70 dB stimulation. In addition, the polar maps show that the green low frequency patch of A1 evoked by 70 dB SPL sound stimuli, is still slightly visible directly after CHL. At 3 d after CHL the activity spots obtained after auditory stimulation with sounds of 60 dB SPL were still absent, however, spots evoked by sounds of 70 dB SPL almost completely recovered predominantly in the LF-fields. Interestingly, LF-fields in the polar maps obtained after auditory stimulation with sweeps of 70 dB SPL always reappeared in blue colors indicating a shift of the cortical responsiveness to higher frequencies.

From the obtained maps we then quantified the sound evoked cortical activity of A1 LF and A1 HF before, directly after and 3 d after CHL (Fig. 2B–G). Quantification showed that the response amplitudes of both A1-fields evoked by sounds of 60 dB SPL significantly decreased to background levels directly after CHL and remained at this level 3 d after CHL (A1 LF response before and after CHL (n = 5): 1.05 × 10^−4^ ± 0.25 × 10^−4^ vs 0.58 × 10^−4^ ± 0.20 × 10^−4^, p = 0.003, paired t-test; A1 LF response before (n = 5) and 3 d after CHL (n = 6): 1.05 × 10^−4^ ± 0.25 × 10^−4^ vs 0.55 × 10^−4^ ± 0.13 × 10^−4^, p = 0.006, unpaired t-test; A1 HF response before and after CHL (n = 5): 1.22 × 10^−4^ ± 0.36 × 10^−4^ vs 0.5 × 10^−4^ ± 0.14 × 10^−4^, p = 0.043, paired t-test; A1 HF response before (n = 5) and 3 d after CHL (n = 6): 1.22 × 10^−4^ ± 0.36 × 10^−4^ vs 0.49 × 10^−4^ ± 0.09, p = 0.002, unpaired t-test; Fig. 2B,C). The background level was defined as the amplitude of the auditory cortex field without auditory stimulation, it always ranged around 0.6 × 10^−4^.

Auditory stimulation with 70 dB SPL also revealed a significant reduction of A1 LF responsiveness directly after CHL (A1 LF response before and after CHL (n = 6): 1.91 × 10^−4^ ± 0.43 × 10^−4^ vs 0.87 × 10^−4^ ± 0.39 × 10^−4^, p = 0.007, paired t-test), however, to a level above background activity. This might indicate that low frequency auditory stimuli of 70 dB SPL can still be transferred into the cochlea after CHL, potentially via bone conduction or sound evoked vibrations of the oval window. Notably, activity in A1 LF elicited by sounds of 70 dB SPL stimulation then strongly increased 3 d after CHL and almost reached the level of animals with intact ears (A1 LF response after CHL (n = 6) vs 3 d after CHL (n = 6): 0.87 × 10^−4^ ± 0.39 × 10^−4^ vs 1.69 × 10^−4^ ± 0.41 × 10^−4^, p = 0.005, unpaired t-test). In contrast, A1 HF activity evoked by 70 dB SPL was decreased to background levels immediately after CHL (A1 HF response before and after CHL (n = 6): 1.78 × 10^−4^ ± 0.54 × 10^−4^ vs 0.48 × 10^−4^ ± 0.16 × 10^−4^, p = 0.004, paired t-test). This might indicate that immediately after CHL, high frequency sounds of 70 dB SPL are no longer transferred into the inner ear, suggesting that these auditory stimuli cannot be conducted by skull vibrations. In addition, responsiveness of A1 HF to sounds of 70 dB SPL did not recover 3 d after CHL (A1 HF response before (n = 6) and 3 d after CHL (n = 6): 1.78 × 10^−4^ ± 0.54 × 10^−4^ vs 0.64 × 10^−4^ ± 0.21 × 10^−4^, p = 0.002, unpaired t-test; Fig. 2D,E). These data suggest that A1 responsiveness is only restored to sound stimuli, which elicited a residual activation of A1 directly after CHL.

In addition, we also observed a recovery of AAF responsiveness evoked by auditory stimulation with 70 dB SPL 3 d after CHL, which does not belong to the primary auditory cortex^32^. However, like in A1, AAF responsiveness to auditory stimuli only increased significantly after 3 days, if we used sound stimuli which evoked a residual activation above background level directly after CHL (70 dB SPL; AAF response to 70 dB SPL before and after CHL (n = 6): 1.82 × 10^−4^ ± 0.42 × 10^−4^ vs 0.63 × 10^−4^ ± 0.20 × 10^−4^, p = 0.003, paired t-test; AAF response to 70 dB SPL after CHL (n = 6) and 3 d after CHL (n = 6): 0.63 × 10^−4^ ± 0.20 × 10^−4^ vs 1.64 × 10^−4^ ± 0.31 × 10^−4^, p = 0.0002, unpaired t-test; Supplementary figure 2).

These indicate that A1 responsiveness to intense deep frequency auditory stimuli with a SPL of 70 dB is almost completely restored 3 d after CHL. In summary, our data suggest an *in vivo* homeostatic mechanism adjusting the stimulus evoked neuronal activity of A1 after afferent input reduction due to CHL.

### Increased mEPSC amplitudes in A1 3 d after CHL

**Figure 3:**
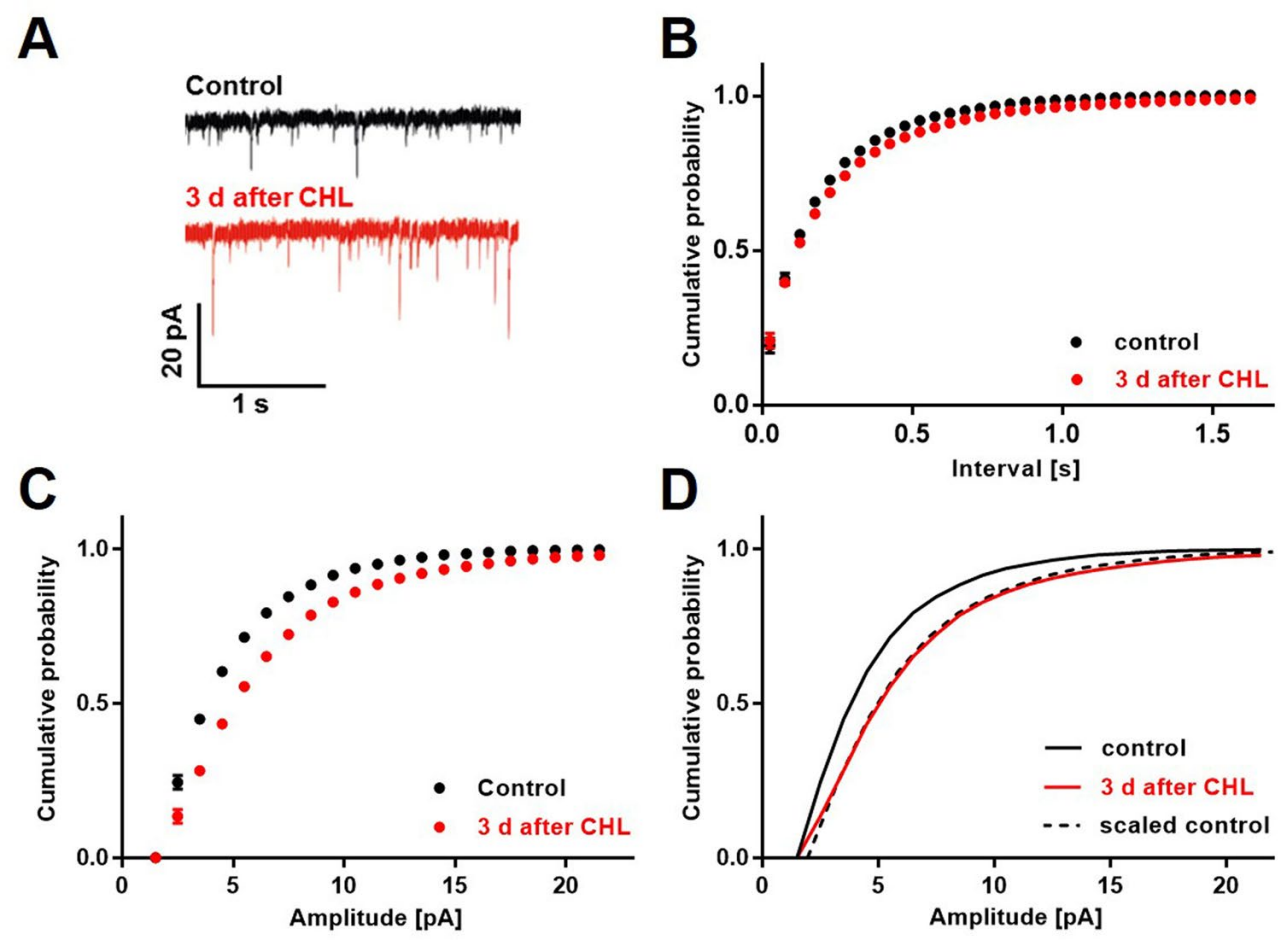
Miniature EPSC amplitude in A1 increased 3 d after CHL. (**A**) Representative traces of mEPSC recordings in layer 2/3 of A1 from one control cell and one cell 3 d after CHL. (**B**) Whole cell recordings in slices revealed that the frequency of mEPSCs remained unchanged 3 d after CHL. (**C**) There was an increase of mEPSC amplitudes in A1 3 d after CHL compared to animals with intact ears (control) (Control and 3 d after CHL: n = 13, N = 3) which led to a right shifted distribution of mEPSC amplitudes. (**D**) Scaling the normalized control distribution of mEPSC amplitudes by the factor 1.3 provided an almost perfect match with the curve obtained 3 d after CHL, indicating a multiplicative synaptic scaling effect.

It has been demonstrated that visual input removal leads to homeostatic plasticity in form of synaptic scaling in the deprived visual cortex, resulting in increased activity levels^9^. Typically, this mechanism is accompanied by an increase of amplitudes of spontaneously occurring mEPSCs^5,7,9,12,37^. To investigate whether synaptic scaling also takes place in A1 after CHL, we performed whole-cell recordings from layers 2/3 pyramids in acute slices from A1 of adult untreated control mice and animals at 3 d after CHL. We did not find changes in mEPSC frequency 3 d after CHL (mEPSC frequency of control mice (13 cells, N = 3) and mice 3 d after CHL (13 cells, N = 3: 4.49 Hz ± 1.36 Hz vs 4.35 Hz ± 2.25 Hz, p = 0.85, unpaired t-test; Fig. 3A,B). However, there was a statistically significant increase of AMPA receptor mediated mEPSC amplitudes in A1 layers 2/3 cells of deprived animals which led to a right shifted mEPSC amplitude distribution, indicating an increased strength of excitatory synapses (mEPSC amplitude of control mice (13 cells, N = 3) and mice 3 d after CHL (13 cells, N = 3): 5.45 pA ± 0.66 pA vs 6.62 pA ± 1.05 pA, p = 0.002, unpaired t-test; Fig. 3A,C). For synaptic scaling it is characteristic that changes in mEPSC amplitudes typically occur in a multiplicative manner^1^. In order to determine whether our observed effect meets this criterion, we scaled the normalized control distribution of mEPSC amplitudes so that it fitted with the measurements obtained 3 d after CHL. Indeed, multiplying the control curve by the factor 1.3 provided an almost perfect match (Fig. 3D). Taken together, these data indicate that CHL induces homeostatic multiplicative synaptic scaling of excitatory synapses in A1 layers 2/3.

### TNFα^−/−^ mice lack recovery of A1 responsiveness 3 d after CHL

Next, we asked whether the scaling effects determined by electrophysiological recordings *in vitro* might contribute to the partial recovery of A1 responsiveness observed by optical imaging *in vivo* 3 d after CHL. To address this issue we measured again the responsiveness of the auditory cortex to sound sweeps of 60 dB and 70 dB SPL in TNFα KO mice with intact ears, directly after CHL and 3 d after CHL and compared it to the WT measurements described above. It has been demonstrated previously that homeostatic plasticity in form of synaptic scaling is deficient in mice lacking the TNFα protein *in vitro* and in the intact visual cortex *in vivo* ^10,24,38^. Thus, we hypothesized that if the recovery of stimulus evoked auditory cortex activation measured by intrinsic imaging might be based on synaptic scaling of cortical layers 2/3 neurons, TNFα deficient mice should lack the recovery of auditory cortex responsiveness 3 d after CHL.

**Figure 4:**
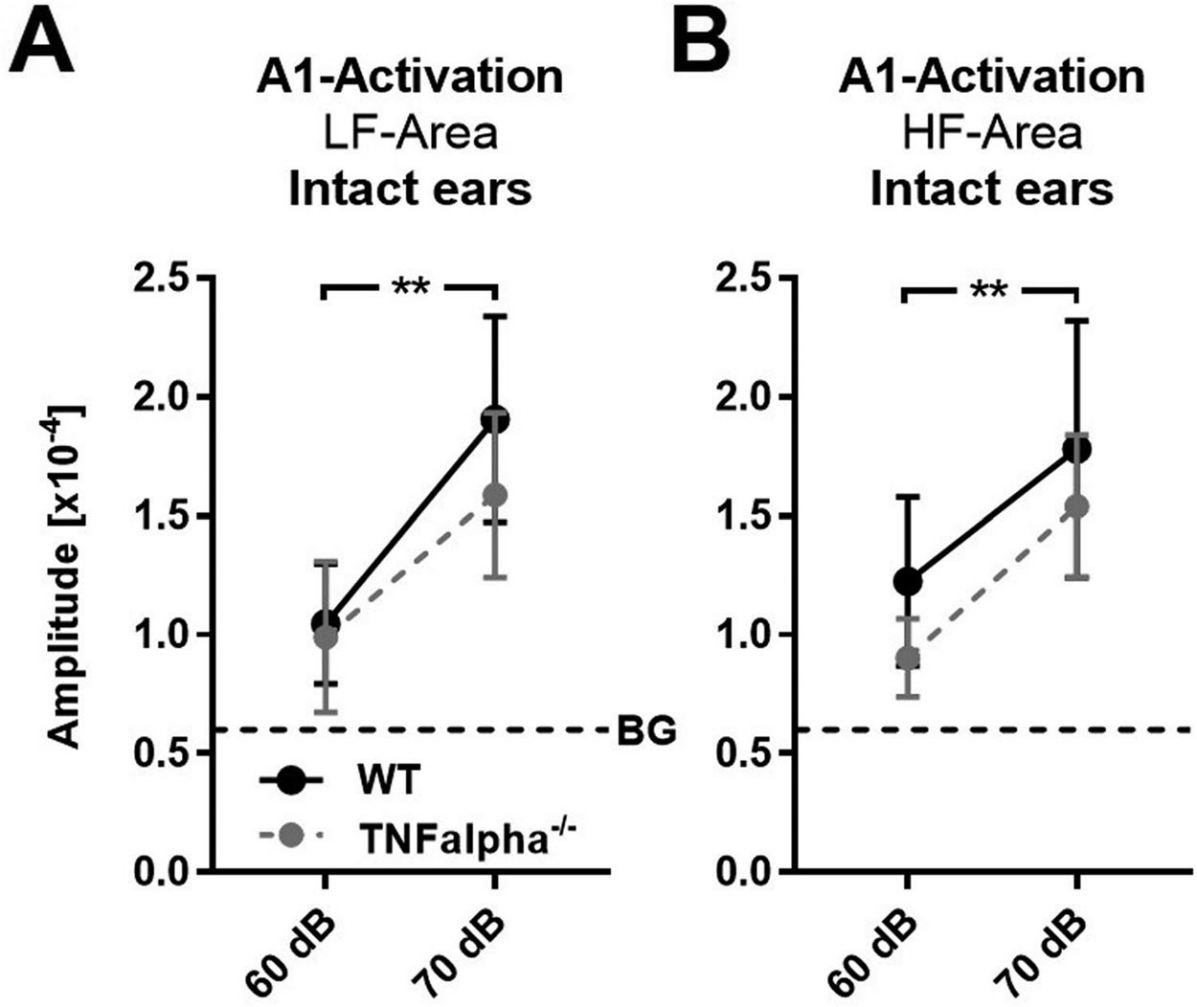
Sound evoked A1 responses are similar in WT and TNFαKO mice with intact ears. (**A**) Activation of A1 LF evoked by auditory stimulation with sound sweeps of 60 dB and 70 dB SPL in WT and TNFαKO mice. (**B**) A1 HF responses elicited by various SPLs in WT and TNFαKO mice. Closed circles represent means ± SD, **p < 0.01, BG: background activation level.

As a first step we compared sound stimulus intensity-response curves of WT (n = 5) and TNFαKO mice (n = 4) with intact ears. Quantification revealed that A1 LF responses evoked by sound sweeps of 60 dB and 70 dB SPL were similar in mice of both genotypes (WT vs TNFαKO, 60 dB: 1.046 × 10^−4^ ± 0.25 × 10^−4^ vs 0.99 × 10^−4^ ± 0.32 × 10^−4^; 70 dB: 1.9 × 10^−4^ ± 0.48 × 10^−4^ vs 1.59 × 10^−4^ ± 0.35 × 10^−4^; F_1,7_ = 1.197, p = 0.31, repeated-measure ANOVA, Fig. 4A). Notably, in both WT and TNFαKO animals there was a marked increase of sound driven A1 LF activity after increasing SPL from 60 dB to 70 dB SPL (F_2,14_ = 7.469, p = 0.006, repeated-measure ANOVA). Furthermore, A1 HF responsiveness to various SPLs was also similar in WT in TNFαKO animals (WT vs TNFαKO, 60 dB: 1.22 × 10^−4^ ± 0.36 × 10^−4^ vs 0.90 × 10^−4^ ± 0.16 × 10^−4^; 70 dB: 1.69 × 10^−4^ ± 0.54 × 10^−4^ vs 1.54 × 10^−4^ ± 0.3 × 10^−4^; F_1,7_ = 0.138, p = 0.72, repeated-measure ANOVA, Fig. 4B). Sound evoked A1 HF activity also significantly increased after increasing the SPL of auditory stimuli from 60 dB to 70 dB in mice of both strains (F_2,14_ = 4.635, p = 0.029, repeated-measure ANOVA). Thus, our data suggest a similar responsiveness of A1 in WT and TNFαKO animals to auditory stimuli of various SPLs and frequencies.

**Figure 5:**
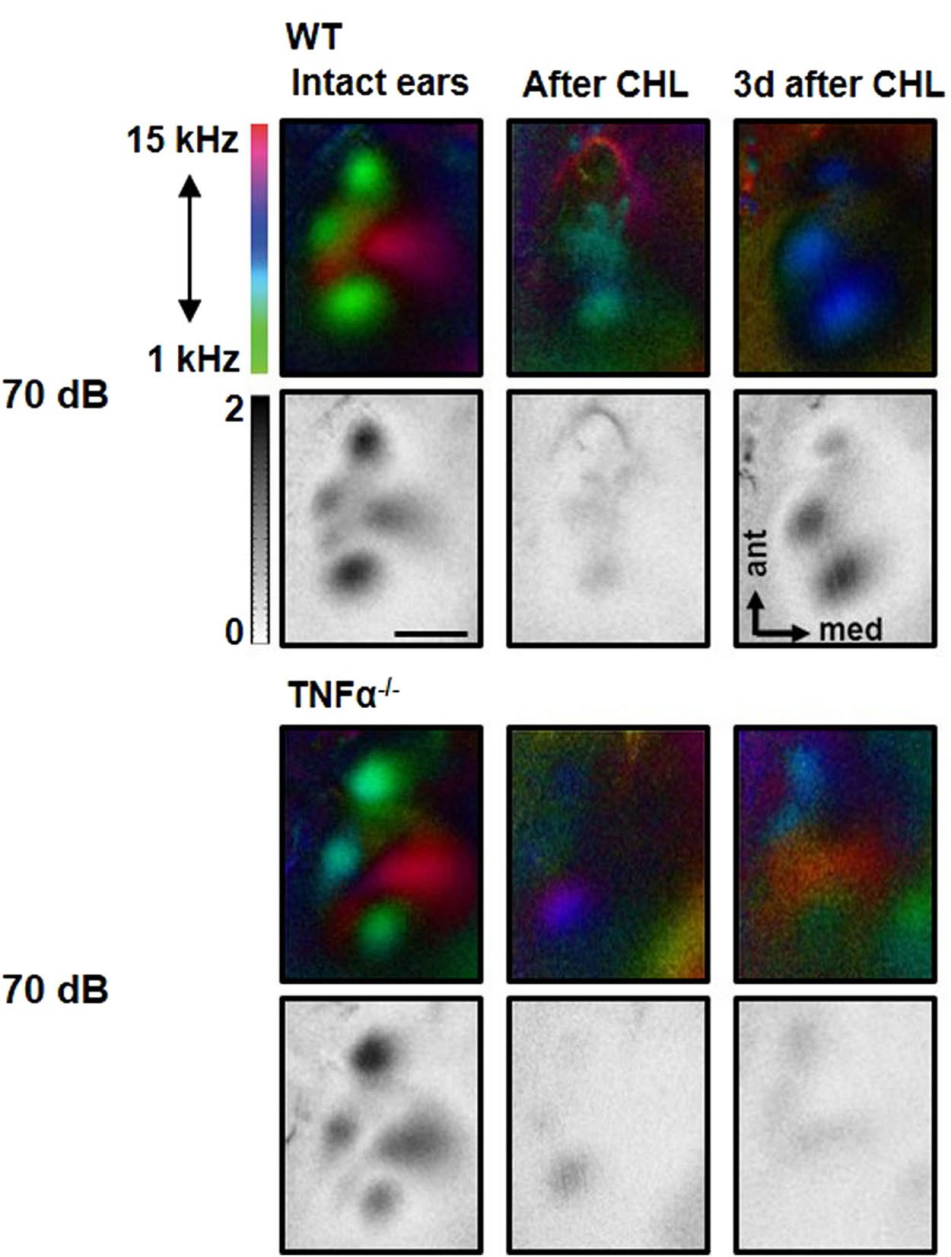
Recovery of evoked auditory cortex activation (A-Activation) is absent in TNFα KO mice. (**A**) Representative color coded polar maps and grey scaled magnitude maps of auditory cortex before and different time points after CHL of WT and TNFα ^−/−^ mice (WT: n = 6; KO: n = 4). Scale bar: 500 µm.

Figure 5 depicts representative sound evoked (70 dB SPL) A1 polar maps and amplitude maps of WT and TNFα^−/−^ mice with intact ears, immediately after and 3 d after CHL. Like in the WT, after bilateral malleus removal the tonotopic map of auditory cortex appeared disorganized and the activity spots were less visible in TNFα^−/−^ animals. This indicates that CHL also leads to a marked reduction of A1 responsiveness to sounds in TNFα deficient mice. However, whereas activity patches reappeared 3 d after CHL in WT mice, they remained almost absent in the KO.

**Figure 6:**
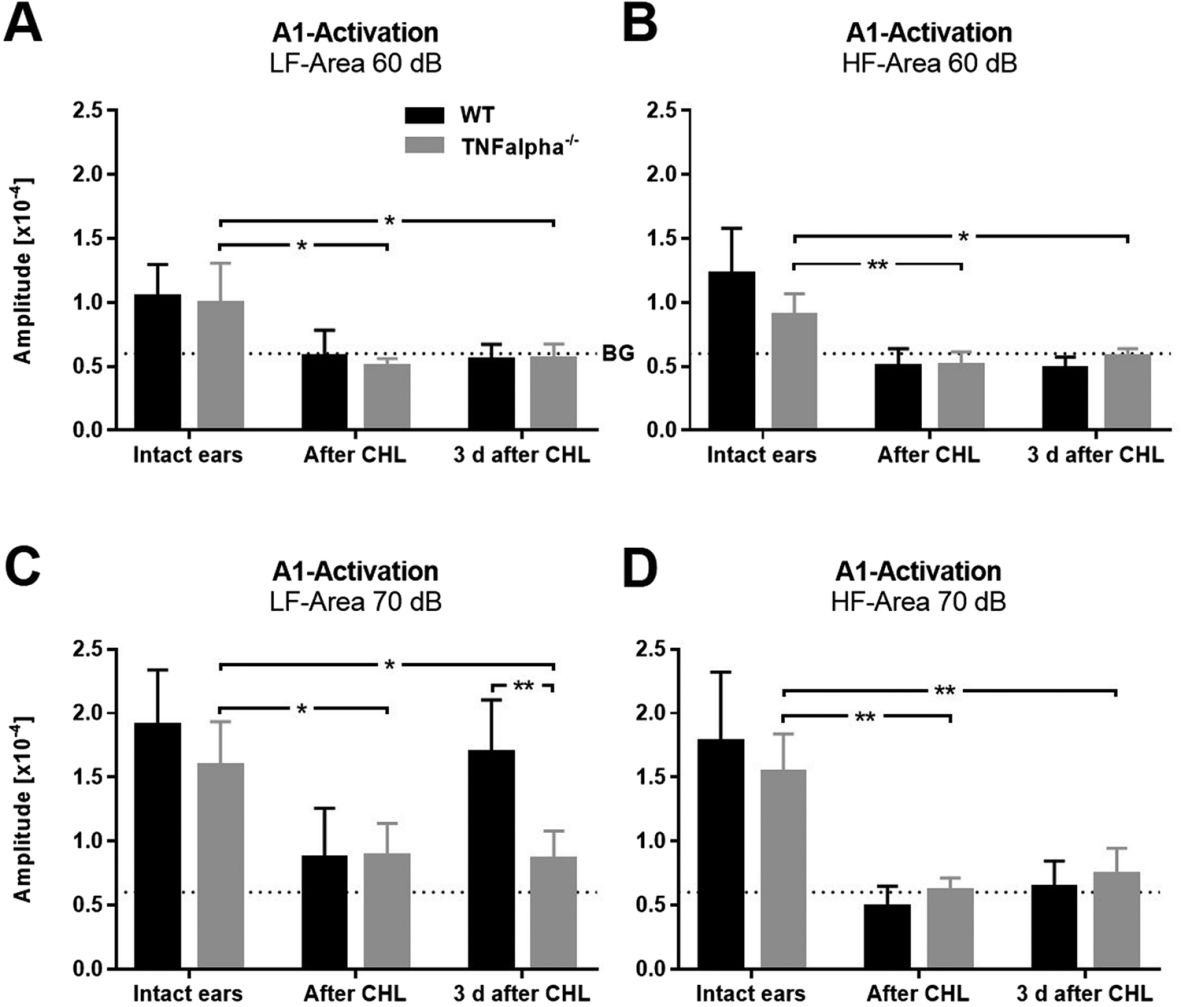
TNFαKO mice lack the recovery of sound driven A1 activation 3 d after CHL. (**A,B**) A1 LF and A1 HF responses evoked by sound sweeps of 60 dB SPL significantly decreased to background levels directly after CHL and remained at this level 3 d after CHL in both WT (intact ears and after CHL: n = 5, 3 d after CHL: n = 6) and TNFαKO mice (intact ears and after CHL: n = 4, 3 d after CHL: n = 4). (**C**) A1 LF responses to auditory stimuli of 70 dB SPL only recovered in the WT (intact ears and after CHL: n = 6, 3 d after CHL: n = 6), but not in the KO (intact ears and after CHL: n = 4, 3 d after CHL: n = 4). (**D**) A1 HF responsiveness to 70 dB SPL decreased directly after CHL and remained at this level after 3 d of CHL in mice of both genotypes (WT, intact ears and after CHL: n = 6, 3 d after CHL: n = 6; TNFαKO, intact ears and after CHL: n = 4, 3 d after CHL: n = 4). Data are presented as means ± SD, *p < 0.05, **p < 0.01.

Quantification showed that A1 LF and A1 HF responses elicited by 60 dB SPL were similar in WT (Intact ears and after CHL: n = 5; 3 d after CHL: n = 6) and TNFα KO (n = 4) mice before, directly after and 3 d after CHL (n = 4) (WT vs KO, all p-values > 0.14, unpaired t-test, Fig. 6A,B): After an initial drop of sound driven A1 activity to background levels due to CHL, A1 responsiveness remained at this level 3 d after CHL in both A1 fields (TNFαKO: A1 LF, intact ears vs after CHL: 0.99 × 10^−4^ ± 0.32 × 10^−4^ vs 0.58 × 10^−4^ ± 0.20 × 10^−4^, p = 0.045, paired t-test; intact ears vs 3 d after CHL: 0.99 × 10^−4^ ± 0.32 × 10^−4^ vs 0.56 × 10^−4^ ± 0.12, p = 0.043, unpaired t-test; A1 HF, intact ears vs after CHL: 0.90 × 10^−4^ ± 0.16 × 10^−4^ vs 0.51 × 10^−4^ ± 0.10 × 10^−4^, p = 0.008, paired t-test; intact ears vs 3 d after CHL: 0.90 × 10^−4^ ± 0.16 × 10^−4^ vs 0.58 × 10^−4^ ± 0.06 × 10^−4^, p = 0.01, unpaired t-test, Fig. 6A,B).

Auditory stimuli of 70 dB SPL also evoked similar A1 LF responses before CHL in mice of both genotypes (p = 0.26, unpaired t-test). Moreover, directly after CHL there was a significant decrease of A1 LF activation to a similar level above background in both WT and KO mice (TNFαKO; A1 LF, intact ears vs after CHL: 1.59 × 10^−4^ ± 0.17 × 10^−4^ vs 0.88 × 10^−4^ ± 0.13 × 10^−4^, p = 0.013, paired t-test, Fig. 6C). However, while A1 LF responsiveness to 70 dB sound stimuli recovered in the WT 3 d after CHL such a recovery was completely absent in KO mice (TNFαKO; intact ears vs 3 d after CHL: 1.59 × 10^−4^ ± 0.35 × 10^−4^ vs 0.86 × 10^−4^ ± 0.22 × 10^−4^, p = 0.012, unpaired t-test; WT vs TNFαKO 3 d after CHL: 1.7 × 10^−4^ ± 0.41 × 10^−4^ vs 0.86 × 10^−4^ ± 0.22 × 10^−4^, p = 0.006, unpaired t-test, Fig. 6C). In addition, responses of A1 HF evoked by auditory stimulation with 70 dB before, immediately after and 3 d after CHL were almost identical in WT and TNFα deficient mice (all p-values > 0.19, unpaired t-test). After a significant decrease of cortical responses to background levels due to CHL A1 HF activity remained at this level 3 d after CHL (TNFαKO; A1 HF, intact ears vs after CHL: 1.54 × 10^−4^ ± 0.30 × 10^−4^ vs 0.62 × 10^−4^ ± 0.10 × 10^−4^, p = 0.004, paired t-test; intact ears vs 3 d after CHL: 1.54 × 10^−4^ ± 0.30 × 10^−4^ vs 0.74 × 10^−4^ ± 0.20 × 10^−4^, p = 0.004 unpaired t-test, Fig. 6D).

In summary, these data suggest that impaired TNFα signaling blocks the recovery of stimulus evoked auditory cortex activity evoked by intense sound stimuli. Moreover, these results suggest that synaptic scaling contributes to the adjustment of A1 responsiveness determined by intrinsic imaging in WT mice 3 d after CHL.

### Recovery of cross-modal function of A1 on V1 along with A1 homeostasis 3 d after CHL

We have previously demonstrated that visually driven V1 activity is suppressed in the presence of a salient sound evoked A1 activation suggesting a cross-modal interplay between A1 and V1^30^. Specifically, using intrinsic signal imaging we could show that reducing sound evoked A1 activity due to CHL induction led to a concomitant increase of visually driven V1 responses. Such an increase of visually evoked V1 activity was also observed in the absence of an concurrent auditory stimulus in animals with intact ears^30^. Hence, we hypothesized that 3 d after CHL, when A1 activity evoked by intense auditory stimuli is back to baseline levels, its suppressive effect on V1 responsiveness might be restored. If so, this would indicate a functional relevance of the homeostatic plasticity in A1.

**Figure 7:**
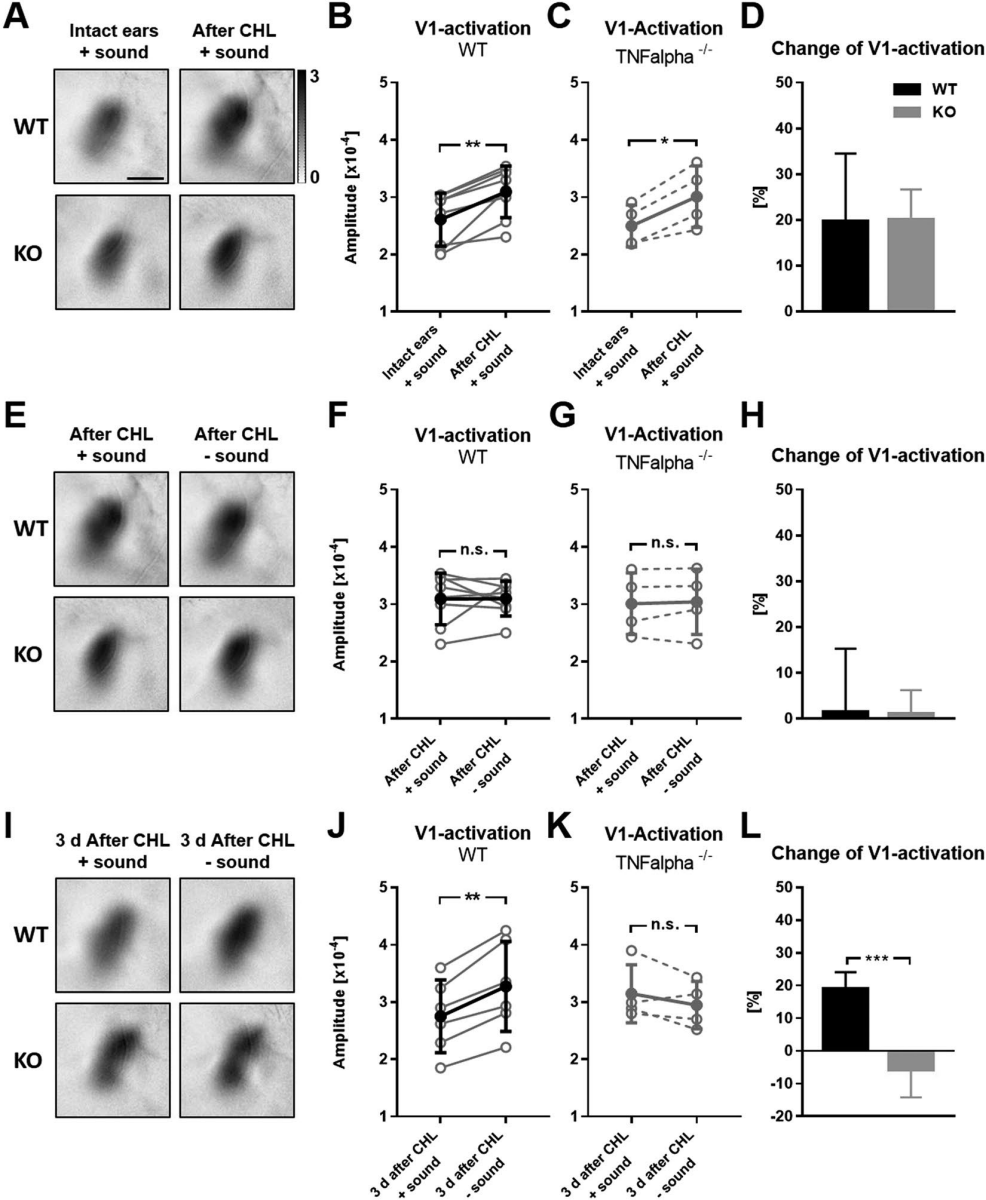
Recovery of the suppressive effect of sounds (70 dB) on visually driven V1 activity 3 d after CHL in WT, but not in TNFαKO mice. (**A**) V1 amplitude maps evoked by binocular visual stimulation under combined auditory stimulation before and directly after CHL of WT (upper row) and TNFαKO mice (lower row). (**B,C**) V1 activity evoked by combined visual and auditory stimulation markedly increased after CHL in the same WT (n = 8) and TNFαKO mice (n = 4). (**D**) Percentage change of visually evoked V1 activity after CHL calculated from B and C. (**E**) V1 amplitude maps obtained after binocular visual stimulation directly after CHL in the presence and absence of sounds of WT (upper row) and TNFα mice (lower row). (**F,G**) Visually driven V1 activity was unchanged after CHL in the presence and absence of sounds in mice of both genotypes (WT, n = 8; TNFαKO, n = 4). (**H**) Percentage change of visually evoked V1 activity in the absence of sounds calculated from F and G. (**I**) V1 amplitude maps of WT (upper row) and TNFαKO mice (lower row) obtained after binocular visual stimulation 3 d after CHL in the presence and absence of sounds. (**J**,**K**) After 3 d of CHL visually evoked V1 activity of WT mice (n = 6) in the presence of sounds was significantly weaker compared to V1 responsiveness in the absence of sounds, whereas V1 responsiveness in TNFαKO mice (n = 4) 3 d after CHL was similar in the presence and absence of sounds. (**L**) Percentage change of visually driven V1 activity in the absence of sounds 3 d after CHL calculated from J and K. Open circles represent measurements of individual animals and closed circles represent the mean ± SD of the open circles. The bars represent the average percentage change of V1 activity ± SD. *p < 0.05, **p < 0.01, ***p < 0.001; Scale bar: 1 mm.

In order to address this presumption we firstly measured visually driven V1 activation under concurrent auditory stimulation (70 dB SPL) before and directly after CHL (10−20 min) in both WT and TNFαKO mice using intrinsic imaging. Figure 7A depicts representative grey-scaled amplitude maps of V1 of mice of both genotypes before and after CHL. It is clearly visible that V1 maps evoked by binocular visual stimulation after CHL were always darker compared to pre CHL conditions. Quantification revealed a marked increase of visually elicited V1 responses after CHL in both WT and TNFαKO mice (WT: 2.61 × 10^−4^ ± 0.46 × 10^−4^ vs 3.09 × 10^−4^ ± 0.45 × 10^−4^, p = 0.002, paired t-test; TNFαKO: 2.5 × 10^−4^ ± 0.37 × 10^−4^ vs 3.01 × 10^−4^ ± 0.54 × 10^−4^, p = 0.015, paired t-test, Fig. 7B,C). In animals of both genotypes we found an increase of visually evoked V1 activity of about 20% after CHL (WT: 19.76% ± 14.73%; TNFαKO: 20.14% ± 6.51%, Fig. 7D). In accordance with our previous study^30^ these data suggest that V1 responsiveness is suppressed by sounds, as long as they evoke a salient auditory cortex activation. However, if sounds only evoke weak A1 responses, like after CHL, visually driven V1 activity increases.

Next, we examined whether auditory stimulation directly (10–20 min) after CHL still affects V1 responsiveness to visual stimuli in the same WT and TNFαKO mice. For this, we measured visually elicited V1 activity directly after CHL in the presence and in the absence of a concomitant sound. As illustrated in Fig. 7E, V1 amplitude maps were equally dark under both conditions described, in mice of both genotypes. Quantification showed that V1 activation evoked by binocular visual stimulation was almost identical in the presence or the absence of sounds after CHL in WT and TNFαKO animals (WT: 3.09 × 10^−4^ ± 0.45 × 10^−4^ vs 3.1 × 10^−4^ ± 0.30 × 10^−4^, p = 0.96, paired t-test; TNFαKO: 3.01 × 10^−4^ ± 0.54 × 10^−4^ vs 3.04 × 10^−4^ ± 0.57 × 10^−4^, p = 0.66, paired t-test, Fig. 7F,G). Hence, we found only subtle changes in V1 responsiveness after CHL in mice of both genotypes (WT: 1.53% ± 13.71%; TNFαKO: 1.0% ± 5.21%, Fig. 7H). Thus, our data suggest that directly after CHL, when A1 responsiveness is dramatically reduced, visually evoked V1 activity is not suppressed by sounds anymore.

As a last step, we measured V1 responsiveness to visual stimuli 3 d after CHL when A1 responsiveness to 70 dB sound stimuli is back to baseline levels in the WT, again, in the presence and absence of a sound stimulus in animals of both genotypes. As depicted in Fig. 7I, V1 maps in the presence of sounds were always weaker than in the absence of sounds in the WT whereas V1 maps of TNFαKO mice were equally dark in both conditions. We found that visually elicited V1 activity was significantly weaker in the presence of sounds compared to V1 activity evoked by visual stimulation without simultaneous auditory stimulation in WT mice (2.75 × 10^−4^ ± 0.64 × 10^−4^ vs 3.27 × 10^−4^ ± 0.79 × 10^−4^, p = 0.001, paired t-test, Fig. 7J). However, in TNFαKO mice visually driven V1 activity was almost equal in the presence and absence of sounds (3.14 × 10^−4^ ± 0.51 × 10^−4^ vs 2.95 × 10^−4^ ± 0.41 × 10^−4^, p = 0.25, paired t-test, Fig. 7K). Quantification of the percentage change of V1 activity in the absence of sound revealed that in WT V1 activity increased by 19.00% ± 5.11% (Fig. 7L) which is, notably, almost identical to the increase of V1 activity immediately after CHL. However, in the KO we found almost no changes of V1 responsiveness in the absence of sounds 3 d after CHL (−5.85% ± 8.36%) leading to a significant difference of V1 activity changes compared to the WT (p = 0.0004, unpaired t-test).

In summary, these data suggest a restoration of the suppressive effects of sounds on V1 responsiveness 3 d after CHL in WT mice, but not in TNFαKO mice. Hence, our results suggest that synaptic homeostatic plasticity reinstates the cross-modal function of A1 on V1 which is typically mediated by an intact A1.

## Discussion

In this study we investigated the effects of a CHL in form of bilateral malleus removal on the neuronal activity of A1 in adult mice. To measure the sound evoked activation of A1, we used optical imaging of intrinsic signals.

We demonstrated that CHL initially led to a marked reduction of A1 responsiveness to sounds which was, however, followed by a complete recovery of A1 activation to low frequency stimuli with a SPL ≥ 70 dB after 3 d *in vivo*. In contrast, A1 responsiveness to high frequency auditory stimulation did only partially recover. Over the same time course, using electrophysiological single unit recordings in slices, we found a multiplicative increase of mEPSC amplitudes in A1 layer 2/3 of the auditory deprived animals, a phenomenon commonly referred to as synaptic scaling^1^. Investigating the effects of a CHL in TNFα deficient mice which lack homeostatic synaptic scaling^10^, showed that recovery of A1 responsiveness was absent in these animals *in vivo*. These results indicate that synaptic scaling contributes to the adjustment of stimulus evoked A1 activation after CHL.

In contrast to SNHL, CHL is characterized by reducing sound transmission into the inner ear without initially affecting the cochlea, thus, representing a mild form of hearing loss^11^. To induce CHL in experimental animals, several approaches have been used. Beside plugging the auditory canal^14–16^ or filling the middle ear cavity using poloxamer^22^, CHL was also induced by malleus removal^11,21,39–41^ as in the present study. In previous studies the effectivity of an experimental induced CHL was typically examined by measuring auditory brainstem responses (ABRs) which massively decreased to all delivered sound intensities and frequencies after this intervention^14,16^. Our approach, using optical imaging of intrinsic signals enabled us to measure sound evoked responses in a high-order processing structures of the auditory pathway – A1 and AAF, before and after CHL. In line with the results obtained by ABR recordings, we found that the responsiveness of A1 markedly decreased after CHL (Fig. 2A–E). Specifically, immediately after CHL A1 activity evoked by sound stimuli of 60 dB SPL was completely abolished. This result is in line with a previous study demonstrating that malleus removal in mice leads to an increase of hearing thresholds, determined by recordings of compound action potentials (CAPs) on the round window, to 50 – 60 dB SPL^42^. However, deep frequency auditory stimuli of 70 dB SPL still evoked weak residual A1 responses (Fig. 2A–E). Taken together, these data indicate that in the range examined here (1 kHz-15 kHz) sounds with higher intensities and lower frequencies most likely reach the auditory pathway after CHL via bone conduction, which is also consistent with ABR recordings^14^.

It has been suggested that homeostatic plasticity adjusts neuronal activity around a stable set-point to compensate perturbations altering neuronal excitability *in vitro* and *in vivo* ^2^. Whereas visual deprivation has been shown to provoke synaptic scaling in V1^7,9^, evidence for synaptic scaling in A1 following an auditory deprivation is rare. SNHL induced by bilateral cochlear ablation results in increased mEPSC amplitudes in A1 layers 2/3 in juvenile mice^12^. However, whether these synaptic changes appear in a multiplicative manner was not examined^12^. In the present study we could show that 3 d of CHL led to multiplicative synaptic scaling in the adult A1 in layers 2/3 (Fig. 3A,C,D). The multiplicative increase of mEPSC amplitudes strongly suggests an insertion of additional AMPARs into the postsynaptic membranes in response to decreased afferent input^6,9^. Similar observations were made in the juvenile and adult visual cortex following visual deprivation^5,9^. In addition, synaptic scaling typically appears 1-3 days after decreasing neuronal activity *in vitro* and *in vivo* ^1,7,9,43^, which is in line with the timing found in the present study. Consistent with recent studies in the visual cortex, we found mEPSC frequency to be unaltered after prolonged sensory deprivation (Fig. 3A,B)^7,9^. These results highlight the similarity of V1 and A1 layers 2/3 neurons in response to sensory deprivation across lifetime. In addition, in line a previous study, our data strongly suggest that the adult A1 retains the ability to undergo experience dependent homeostatic synaptic plasticity into adulthood^13^.

Most earlier studies investigated homeostatic effects after sensory deprivation only on spontaneous spiking of single cells in slices^5,7,9,37,44^ or on spontaneous activity of V1 neurons *in vivo* ^9^. However, in the present study, using optical imaging, we were able to measure stimulus evoked activity in the deprived cortex after a prolonged activity reduction *in vivo*. We found a complete recovery of A1 LF responsiveness to sound sweeps at 70 dB SPL 3 days after the induction of CHL (Fig. 2A,D). This result is consistent with previous studies in V1 demonstrating an adjustment of visually evoked responses after prolonged visual deprivation due to homeostatic mechanisms^10,45^. Likewise, SNHL led to an increased excitability of A1 layers 2/3 neurons in A1 slices of juvenile animals^12^. These data indicate that homeostatic plasticity does not only adjust spontaneous neuronal activity, but also adjusts cortical responsiveness to reduced afferent input by scaling up postsynaptic responses in layers 2/3.

Is the partial restoration of evoked A1 activation a functional consequence of synaptic scaling induced by CHL? Previous *in vitro* studies could demonstrate that blocking TNFα signaling before or during a TTX induced neuronal activity blockade of cultured pyramidal neurons prevents synaptic scaling^24,38^. Moreover, synaptic scaling is absent in mice lacking the TNFα protein *in vivo* ^10^. Since there was no recovery of A1 responsiveness in TNFα KO mice after CHL in the present study (Figs 5 and 6), we conclude that the restoration of A1 activation evoked by sounds requires an intact TNFα signaling. Therefore, our results suggest that TNFα, and hence, synaptic scaling contributes to the homeostatic adjustment of evoked A1 activity after a prolonged sensory deprivation in adults.

Additionally, since the present study focused on the effects of CHL in A1, we cannot exclude whether synaptic scaling or other homeostatic mechanisms take place at lower-order stages of the auditory pathway, which is, however, discussed controversially^16,46^.

We found that the suppressive effect of salient sound evoked A1 responses on visually driven V1 activity was precisely restored along with TNFα dependent A1 homeostasis (Fig. 7). In other words, synaptic scaling in A1, induced by CHL, restored the cross-modal effects of A1 on V1, which are typically mediated by an intact A1^30,47^. These data suggest that synaptic scaling acts to restore the precise function of neuronal networks without losing information. This conjecture is in line with the fact that synaptic scaling globally increases or decreases the strength of all synapses in a multiplicative manner, allowing the conservation of information because the difference in synaptic weights remains preserved^1,4^.

In summary, our findings strongly suggest that synaptic scaling contributes to the stimulus evoked recovery of A1 activity after CHL. In addition, we show that changes of auditory experience can massively affect neuronal properties in A1 even in adult mice far beyond their sensory critical periods^22^. In future, it might be interesting to investigate whether the recovery of stimulus evoked A1 activation after CHL creates a behaviorally relevant perception which would indicate a recovery of cortical hearing. If so, this effect would have important implications for individuals suffering from CHL.

## Materials and Methods

### Animals

Since it has been described that the commonly used C57BL/6 JOlaHsd mice lack homeostatic synaptic scaling^45^, we did not use mice of this strain. C57BL/6J (Jackson labs), which are shown to display synaptic scaling^45^ and TNFαKO mice (shown to lack synaptic scaling *in vivo* ^10^) were raised in standard cages on a 12 h light/dark cycle, with food and water available *ad libitum*. Animal housing in our institution is regularly supervised by veterinaries from the state of Thuringia, Germany. For the present study we used fully adult male mice (P90-P150). All experimental procedures have been performed according to the German Law on the Protection of Animals and the corresponding European Communities Council Directive of November 24, 1986 (86/609/EEC), and were approved by the Thüringer Landesamt für Lebensmittelsicherheit und Verbraucherschutz (Thuringia State Office for Food Safety and Consumer Protection) under the registration number 02-050/14 and 02-032/16. For the present study we used a total number of 18 C57BL/6 J and 8 TNFαKO mice.

### Induction of CHL

A CHL was always induced by bilateral malleus removal as described previously^11^. Animals were initially anesthetized with 4% isoflurane. During the surgery the anesthesia was maintained at 2% isoflurane in a mixture of 1:1 O_2_/N_2_O applied through a plastic mask. Additionally, mice received a subcutaneous injection of carprofene (4 mg/kg) for pain prevention. One carprofene injection was administrated daily after CHL. The eyes of the animal were protected with silicon oil. The tympanic membrane was punctured and the malleus was removed under visual control through this opening using fine sterilized forceps^11,21,30^. Great care was taken to avoid any destruction of the stapes and the oval window which is visible through the hearing canal (see^21^). After the surgery animals were returned to their home cages for 3 d. Animals received a daily injection of carprofene (4 mg/kg).

### Mouse preparation for optical imaging

Animals were initially anesthetized with 4% isoflurane in a mixture of 1:1 O_2_/N_2_O and placed on a heating blanket (37.5 °C) for maintaining body temperature. Subsequently, mice received an injection of chlorprothixene (40 µg/mouse i.m.) and carprofen (4 mg/kg). The inhalation anesthesia was applied through a plastic mask and maintained at 0.5% during the experiment. We continuously recorded the respiration rate and heart rate in order to analyze anesthesia level during intrinsic optical imaging using BIOPAC. The skin above the right hemisphere was removed. Using dental acrylic a metal bar was glued to the skull to fix the animal in a stereotaxic frame. Next, we exposed the skull overlying the left auditory cortex. The exposed area was covered with 2.5% agarose in saline and sealed with a glass coverslip. Cortical responses were always recorded through the intact skull.

### Optical imaging of intrinsic signals

#### Imaging of auditory cortex

Responses of mouse auditory cortex were recorded based on the intrinsic imaging method described previously^25,29,30^. Briefly, the method uses a periodic stimulus that is presented to the animal for some time and cortical responses are extracted by Fourier analysis. In our case the auditory stimulus was a tone sweep linearly ascending or descending in frequency in the range of 1 kHz to 15 kHz with 60 dB or 70 dB sound pressure level (SPL) and a temporal frequency of 0.125 Hz. Sound stimuli were designed using Audacity software and were delivered by free field speakers placed 20 cm next to both ears and presented for 5 min in each run. In each imaging session two images of the cortical response to auditory stimulation to each direction (ascending or descending) were taken. Background activity of A1 was measured in the region of A1 without preceding sound stimulation. Calculation of background activity was performed in the same way like A1 map analysis (see Data analysis). We always measured A1 activity evoked by bilateral auditory stimulation.

#### Imaging of the visual cortex

To study visually driven V1 activity in the absence of sounds, responses of mouse visual cortex were recorded as originally described by Kalatsky and Stryker (2003). In our case, the visual stimulus was a drifting horizontal light bar of 2° width, a spatial frequency of 0.0125 cycles/degree (cyc/deg), 100% contrast and a temporal frequency of 0.125 Hz. The stimulus was presented on a high refresh rate monitor (Hitachi Accuvue HM 4921-D) placed 25 cm in front of the animal. Visual stimulation was adjusted so that it only appeared in the binocular visual field of the recorded hemisphere (−5° to + 15° azimuth, −17° to + 60° elevation). The stimulus was presented to both eyes for 5 min. Thus, the stimulus was repeated for about 35 times during one presentation. We always measured V1 activity elicited by binocular visual stimulation.

#### Combined auditory and visual stimulation

To investigate the cross-modal effects of sounds on visually driven V1 activity before, after and 3 d after CHL the sound sweep described above (70 dB, 1 kHz – 15 kHz) was synchronized with the visual stimulus (moving light bar). In detail, as the bar started moving from the bottom of the monitor (−15°), the tone started at the same time with its lowest frequency (1 kHz). During the following 8 s the bar moved to the top of the monitor while the tone ascended linearly in frequency to 15 kHz. The synchronization was also maintained after the stimulus reversal.

#### Dalsa CCD camera recording procedure

Using a Dalsa 1M30 CCD camera (Dalsa, Waterloo, Canada) with a 135 × 50 tandem lens (Nikon, Inc., Melville, NY) we first recorded images of the surface vascular pattern via illumination with green light (550 nm) and, after focusing below 600 μm below the cortical surface, the intrinsic signals were obtained under illumination with red (610 nm) light. Frames were acquired at a rate of 30 Hz and temporally averaged to 7.5 Hz. The obtained 1024 × 1024 pixel images were spatially averaged to a 512 × 512 resolution.

#### Induction of CHL between two imaging sessions

To measure the stimulus evoked A1 and V1 responsiveness before and after CHL in the same animals, we performed one imaging session with intact ears and one session subsequently after CHL. After the first session the anesthetized animal was removed from the stereotaxic frame. The eyes of the animal were protected with silicon oil. Malleus was removed under visual control using fine sterilized forceps as described above. After this surgery the animal was re-fixed at the stereotaxic frame as described above and the CCD imaging camera was adjusted at the same position (like in the first session) above the region of interest guided by the vascular pattern. Subsequently (after 10–20 min), the next imaging session was started.

#### Data analysis

From the recorded frames the signal was extracted by Fourier analysis at the stimulation frequency and converted into amplitude and phase maps using custom software^25^. For data analysis we used MATLAB^48^. In detail, from a pair of the upward and downward (ascending or descending, respectively) maps, a map with absolute visoutopy or tonotopy and an average magnitude map was computed. The magnitude component represents the activation intensity of the visual or auditory cortex. All magnitudes were multiplied by 10^4^ so that they can be presented in small numbers. To each condition (before or after CHL) we took at least three magnitudes of V1 or A1 responsiveness and averaged them for data presentation.

### Electrophysiology

#### Slice preparation

350-µm-thick brain slices were prepared from 3-month-old C57BL/6 J control mice and 3 d after CHL induction and equilibrated in aCSF (in mM): 120 NaCl, 3 KCl, 1.3 MgSO_4_, 1.25 NaH_2_PO_4_, 2.5 CaCl_2_, 10 D-glucose, 25.0 NaHCO_3_, gassed with 95% O_2_ / 5% CO_2_, pH 7.3 at room temperature for at least 1 h, as described previously^49^.

#### Patch Clamp recordings

Coronal brain slices were placed in a submerged recording chamber mounted on an upright microscope (BX51WI, Olympus). Slices were continuously superfused with aCSF (2–3 ml/min, 32 °C, pH 7.3). Patch clamp recordings of miniature excitatory postsynaptic currents (mEPSCs) were performed in pyramidal neurons as described previously^49^. Neurons in A1 were selected for recording if they displayed a pyramidal-shaped cell body, in agreement with the morphology of principal neurons in the mouse cortex. Interneurons, which are usually smaller and exhibit very high input resistance values, were avoided^50^. mEPSCs were recorded at a holding potential of −70 mV for at least 5 min in aCSF. mEPSCs were isolated by adding tetrodotoxin (0.5 μm, Tocris Bioscience) and bicuculline methiodide (20 μM, Biomol) to block action potential-induced glutamate release and GABA_A_ receptor-mediated mIPSCs, respectively. 30 μM (2 *R*)-amino-5-phosphonovaleric acid (dl-APV; Sigma-Aldrich) was added to suppress NMDA currents. The pipette solution contained the following (in mM): 120 CsMeSO_4_, 17.5 CsCl, 10 HEPES, 5 BAPTA, 2 Mg-ATP, 0.5 Na-GTP, 10 QX-314 [*N*-(2,6-dimethylphenylcarbamoylmethyl) triethylammonium bromide], pH 7.3, adjusted with CsOH. Data analysis was performed off-line with the detection threshold levels set to 3 pA for mEPSCs. The following parameters were determined: frequency and peak amplitude.

### Statistical analysis

Optical imaging data obtained from the same mice before and immediately after CHL were compared using a paired *t-*test. Optical imaging data obtained 3 d after CHL were compared with data obtained before and directly after CHL using an unpaired *t*-test. P values were then Bonferroni corrected. Electrophysiological data were compared using an unpaired *t-*test. Comparison of imaging data of WT and KO animals was performed using a repeated-measure ANOVA or an unpaired *t-*test. The level of significance was set as *p < 0.05; **p < 0.01; ***p < 0.001. Data are presented as means and standard deviation (SD) of the mean or as measurements of individual animals.

### Data availability

The datasets generated and analyzed within the present study are available from the corresponding author on reasonable request.

## Electronic supplementary material

Supplementary information
