# Supplementary material for: Homeostatic plasticity and synaptic scaling in the adult mouse auditory cortex
Source: Sci Rep. 2017 Dec 12;7:17423. doi: 10.1038/s41598-017-17711-5 (PMC5727212; doi:10.1038/s41598-017-17711-5)
Supplement: Supplementary file 1 — Supplementary information [file 41598_2017_17711_MOESM1_ESM.pdf]

Homeostatic plasticity and synaptic scaling in the adult mouse auditory cortex

Running title: Homeostatic plasticity in the auditory cortex

Manuel Teichert[1], Lutz Liebmann[2], Christian A. Hübner[2] and Jürgen Bolz[1*]

[1]University of Jena, Institute of General Zoology and Animal Physiology, 07743 Jena, Germany

[2]University of Jena, University Hospital Jena, Institute of Human Genetics, 07743 Jena, Germany

***Corresponding author:**

Name:	Jürgen Bolz
Address:	Erbertstraße 1, 07743 Jena; Germany
Email:	jurgen.bolz@uni-jena.de
Tel.:	0049 03641 49101
Facsimile number:	0049 03641/949102

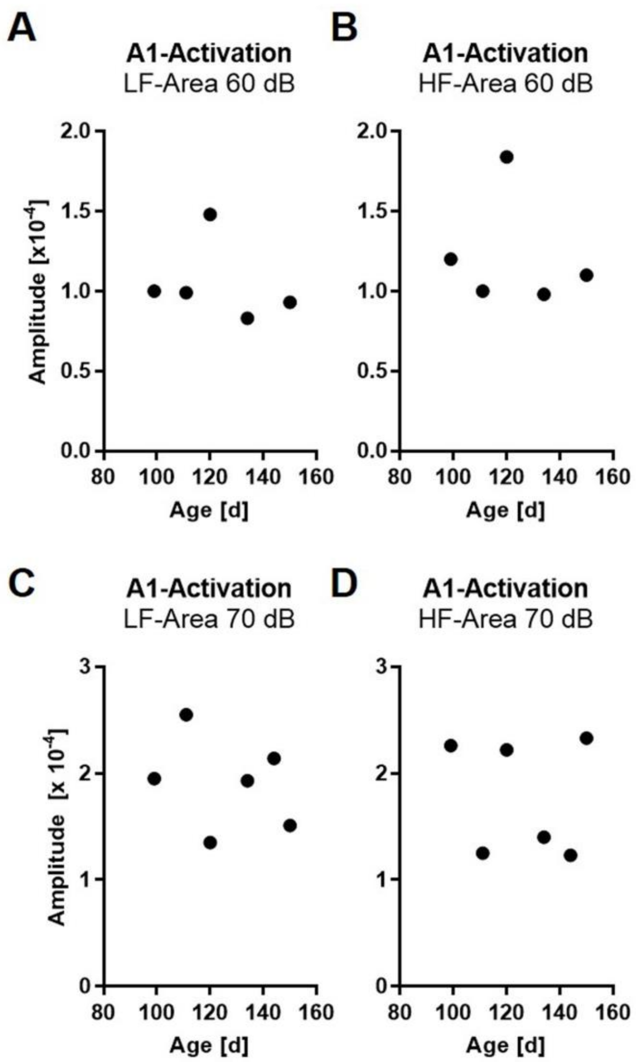

Supplementary Figure 1: No age related changes of sound evoked A1 responses. (A, B, C, D) A1 LF and A1 HF activity evoked by sounds at 60 dB and 70 dB SPL was blotted against the age of the animals. Filled circles represent measurements of individual animals (60 dB, n = 5; 70 dB, n = 6).

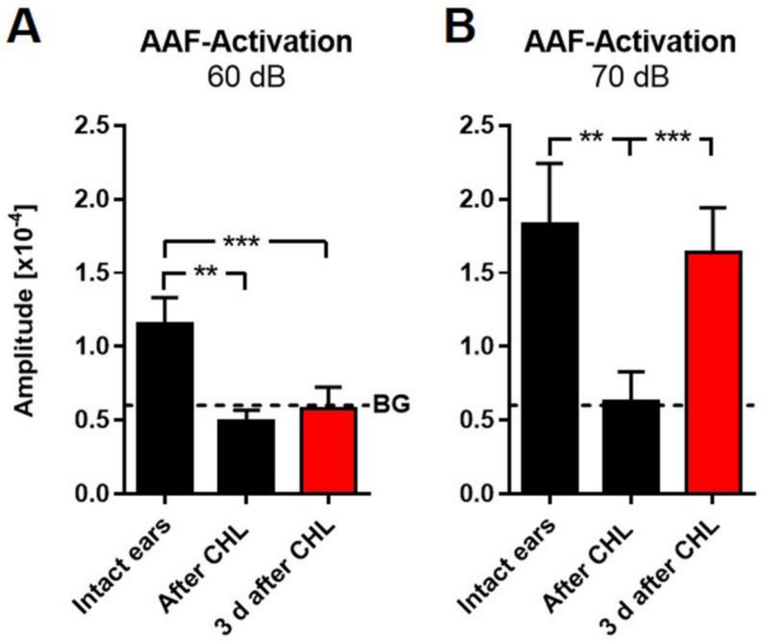

Supplementary Figure 2: Recovery of evoked AAF responsiveness 3 d after CHL revealed by intrinsic signal imaging. (**A**) Responsiveness of the AAF to auditory stimuli of 60 dB SPL was completely abolished directly after CHL and remained at this level at 3 d after CHL (intact ears and after CHL: $n = 5$; 3 d after CHL: $n = 6$). (**B**) Activity levels of AAF evoked by sound sweeps of 70 dB SPL were also reduced after CHL (70 dB, intact ears and after CHL: $n = 6$). However, sound evoked activation significantly recovered 3 d after CHL (70 dB, 3 d after CHL: $n = 6$). Data are presented as means ± SD, $*p < 0.05$, $**p < 0.01$, $***p < 0.001$
